# Biofilm Signaling, Composition and Regulation in *Burkholderia pseudomallei*

**DOI:** 10.4014/jmb.2207.07032

**Published:** 2022-10-17

**Authors:** Pravin Kumran Nyanasegran, Sheila Nathan, Mohd Firdaus-Raih, Nor Azlan Nor Muhammad, Chyan Leong Ng

**Affiliations:** 1 Institute of Systems Biology, Universiti Kebangsaan Malaysia, 43600 UKM Bangi, Selangor, Malaysia; 2 Faculty of Science and Technology, Universiti Kebangsaan Malaysia, 43600 UKM Bangi, Selangor, Malaysia

**Keywords:** *Burkholderia pseudomallei*, biofilm, exopolysaccharide, eDNA, cyclic-di-GMP, quorum sensing

## Abstract

The incidence of melioidosis cases caused by the gram-negative pathogen *Burkholderia pseudomallei* (BP) is seeing an increasing trend that has spread beyond its previously known endemic regions. Biofilms produced by BP have been associated with antimicrobial therapy limitation and relapse melioidosis, thus making it urgently necessary to understand the mechanisms of biofilm formation and their role in BP biology. Microbial cells aggregate and enclose within a self-produced matrix of extracellular polymeric substances (EPSs) to form biofilm. The transition mechanism of bacterial cells from planktonic state to initiate biofilm formation, which involves the formation of surface attachment microcolonies and the maturation of the biofilm matrix, is a dynamic and complex process. Despite the emerging findings on the biofilm formation process, systemic knowledge on the molecular mechanisms of biofilm formation in BP remains fractured. This review provides insights into the signaling systems, matrix composition, and the biosynthesis regulation of EPSs (exopolysaccharide, eDNA and proteins) that facilitate the formation of biofilms in order to present an overview of our current knowledge and the questions that remain regarding BP biofilms.

## Introduction

*Burkholderia pseudomallei* (BP) is a gram-negative, environmental saprophyte predominately found in the soil and surface groundwater of endemic tropical and subtropical regions worldwide [1, 2]. BP is the etiological agent of melioidosis, a life-threatening disease that accounts for approximately 89,000 deaths per year worldwide [2-6]. Diabetes mellitus remains a major risk factor for melioidosis; therefore, the rising global diabetes pandemic could further escalate the number of deaths attributed to melioidosis [2]. The virulence factors of BP include lipopolysaccharide (LPS), flagella, capsule, and type III secretory systems (TTSS), which have been identified to be involved in acute septicaemia and chronic melioidosis [1, 7]. These virulence factors enhance bacterial survival and persistence across a wide range of hosts and facilitate evasion of the host’s immune response [1, 7]. In addition, BP isolates that can form biofilm have been associated with the relapse of melioidosis, and BP within the biofilm community is more resistant to antibiotics [8].

**Fig. 1:**
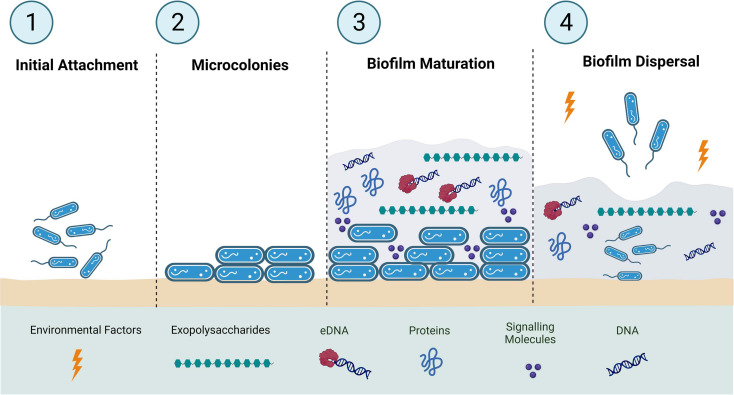
Schematic diagram representing four stages of biofilm formation (1) surface bacterial attachment, (2) microcolony formation, (3) maturation of biofilm architecture, and (4) dispersion of cells into the planktonic state (adapted from [15-17]).

Many bacterial pathogens are known to form biofilm, which encloses the bacteria and facilitates cellular attachment and interaction [9]. In addition, the biofilm also renders the pathogen more tolerant to antibacterial agents and host immune molecules while aiding bacterial survival under nutrient-deficient conditions [10, 11]. Bacterial biofilms are composed of an aggregation of microbial cells on biotic or abiotic surfaces enclosed by a self-produced matrix of extracellular polymeric substances (EPSs) composed of proteins, polysaccharides, nucleic acids (DNA), lipopolysaccharides (LPS), and water [10-14]. A successful biofilm formation involves four main stages: (i) surface bacterial attachment, (ii) microcolony formation, (iii) maturation of biofilm architecture, and (iv) signals and environmental cues that trigger the dispersion of cells into the planktonic state [15-17]. The common biofilm formation processes in microbes are illustrated in Fig. 1.

In BP, biofilm formation is closely associated with its ability to adapt or survive in various environmental niches, as well as contributing to the bacteria’s pathogenicity [18, 19]. The emergence of resistance to antibiotics, including ceftazidime (CTZ), doxycycline (DOX), and imipenem, which are common drug treatments against melioidosis, is generally attributed to the presence of biofilm surrounding BP [20-22]. Despite the importance of biofilm formation features in BP that are linked to clinical pathogenicity and virulence, the detailed mechanism of biofilm formation in BP is yet to be elucidated. For the past decade, researchers have utilized genetics and ‘omics’ approaches targeting the biofilm biosynthesis pathway of BP in studies that have successfully identified genes and proteins that are crucial for BP biofilm formation. In addition, studies on other *Burkholderia* species that share high genome sequence similarity to BP have provided indirect evidence that further helps elucidate BP biofilm formation. Through this review, we aimed to provide current insights into EPS and biofilm formation in BP and highlight the potential genes, proteins, and pathways that warrant further investigation to develop effective therapeutics or successful vaccine candidates to treat melioidosis.

## Signaling Systems That Promote BP Biofilm Formation

Environmental factors are known to trigger the formation or dispersal of biofilm in most bacteria [23, 24]. Environmental cues such as temperature, pH, nutrient deficiency, and glucose were reported to influence biofilm formation by BP [25-27]. These ecological factors are sensed by signaling molecules, which can influence gene expression in support of biofilm formation and facilitate the conversion of free-living planktonic cells into biofilm cells [25, 28]. Cyclic di-GMP (c-di-GMP), quorum-sensing (QS) molecules, and small RNAs (sRNAs) are known to be the major signaling molecules present in the biofilm community [29-31]. c-di-GMP signaling occurs during the early stages of biofilm formation to facilitate the conversion of free-living planktonic cells to biofilm cells, while QS signaling is involved during biofilm maturation and dispersion [29, 32-34]. sRNAs serve as regulatory molecules in several bacterial metabolic processes, including *Burkholderia* species for biofilm development [31, 35]; nonetheless, biofilm-associated sRNAs in BP have yet to be identified.

## Cyclic-di-GMP Signaling

C-di-GMP is a bacterial universal intracellular secondary signaling molecule [36-38]. In bacterial biofilm formation, c-di-GMP is known to regulate genes responsible for synthesizing EPS components; extracellular polymeric exoenzymes, polysaccharides, and adhesins [39, 40]. In addition, c-di-GMP enhances bacterial adhesion and represses bacterial motility, further promoting biofilm production [32, 33, 41, 42]. Furthermore, depletion of c-di-GMP levels has been reported to trigger the dispersal of biofilms. For instance, inhibition of the final step of the denitrification pathway has been implicated in inducing biofilm dispersal [43]. Nitrate levels have been reported to significantly affect biofilm formation in BP, as they ultimately determine the fate of c-di-GMP [33]. The denitrification process, which involves the reduction of nitrate to nitrogen, is important in BP biofilms as it provides an alternative energy source under oxygen-limited conditions [33, 44]. The impact of inhibiting the denitrification pathway on biofilms was recently evaluated in *B. thailandensis*, a species closely related to BP [43]. Inhibiting the final step of denitrifying nitrous oxide to nitrogen catalyzed by nitrous oxide reductase leads to the accumulation of nitrous oxide, which in turn reduces c-di-GMP levels that ultimately trigger the dispersal process [43, 45]. As for BP, a recent transcriptome analysis between high and low BP biofilm-forming isolates revealed the overexpression of nitrous oxide reductase, *bpsl1607*, in the high biofilm-forming isolate. Furthermore, studies on BP 1026b isolate mutants involving a two-component, nitrate-sensing system in the form of *narX-narL* (equivalent to *bpsl2313*-*bpsl2314*) have further confirmed the regulation of the denitrification pathway in c-di-GMP production and biofilm formation [44].

The synthesis and breakdown of c-di-GMP in most bacteria are regulated by diguanylate cyclase (DGC) and phosphodiesterase (PDE), respectively. The activity of both proteins is affected by environmental cues, in agreement with the transition of bacteria from planktonic to biofilm state being regulated by c-di-GMP in response to changes in environmental stimuli [46-48]. DGC contains the conserved GGDEF domain, while PDE contains a conserved EAL or HD-GYP domain [11, 49]. DGC catalyzes the synthesis of c-di-GMP from the condensation of two GTP molecules, while PDE catalyzes the hydrolysis of c-di-GMP, resulting in two GMP molecules [50, 51]. *Burkholderia cenocepacia* is another *Burkholderiaceae* family member and closely related species to BP, and a number of genes encoding proteins that are homologous across the *Burkholderia* group responsible for the synthesis of c-di-GMP in *B. cenocepacia* have been identified and tabulated (Table 1) [51].

In BP, a putative DGC (*bpss2342* or *Bp1026b_II2523*) that contains a conserved GGDEF domain was reported to influence the biofilm formation in a temperature-dependent manner in which increased biofilm formation was observed among mutant colonies grown at 37°C compared to 30°C [41, 52]. This observation highlights the correlation between c-di-GMP synthesis and environmental factors regulating BP's biofilm formation. Furthermore, the *Bp1026b_II2523* (*bpss2342*) mutant was shown to affect various biological systems such as polysaccharide biosynthesis and several secretion systems (T3SS-3, T3SS-2, T6SS-3, and T6SS-6) and biosynthetic gene clusters (BGCs) that are involved in non-ribosomal peptide and polyketide synthesis and, predicted to encode small metabolites contributing to biofilm development [52]. Apart from that, the *cdpA* gene in BP KHW (corresponding to *bpsl1263* in BP K96243) encoding phosphodiesterase proteins that contains a conserved EAL has been identified as PDE [42]. The *cdpA* deletion mutant was shown to exhibit high levels of c-di-GMP which favor biofilm production through increased exopolysaccharide and cellular aggregation [42]. Furthermore, higher expression of the *cdpA* gene was observed for BP exposed to exogenous sodium nitrate. This led to the upregulation of PDE activity which contributed to reduced c-di-GMP levels and, subsequently, poor biofilm formation [33]. Recently, a c-di-GMP signaling cascade mediated by a pdcABC operon that can regulate virulence, motility, and biofilm formation was reported for *B. thailandensis* [53]. pdcA encodes a DGC protein that produces c-di-GMP and is regulated by PdcC (phosphate-accepting response regulator). The phosphorylated PdcC inhibits PdcA by binding to its PAS domain. PdcB is a phosphatase that increases the activity of PdcA through dephosphorylation of PdcC [53]. Interestingly, an operon in BP shares high sequence identity with pdcABC (Table 1), suggesting that BP may share a similar pathway in modulating c-di-GMP levels.

**Fig. 2:**
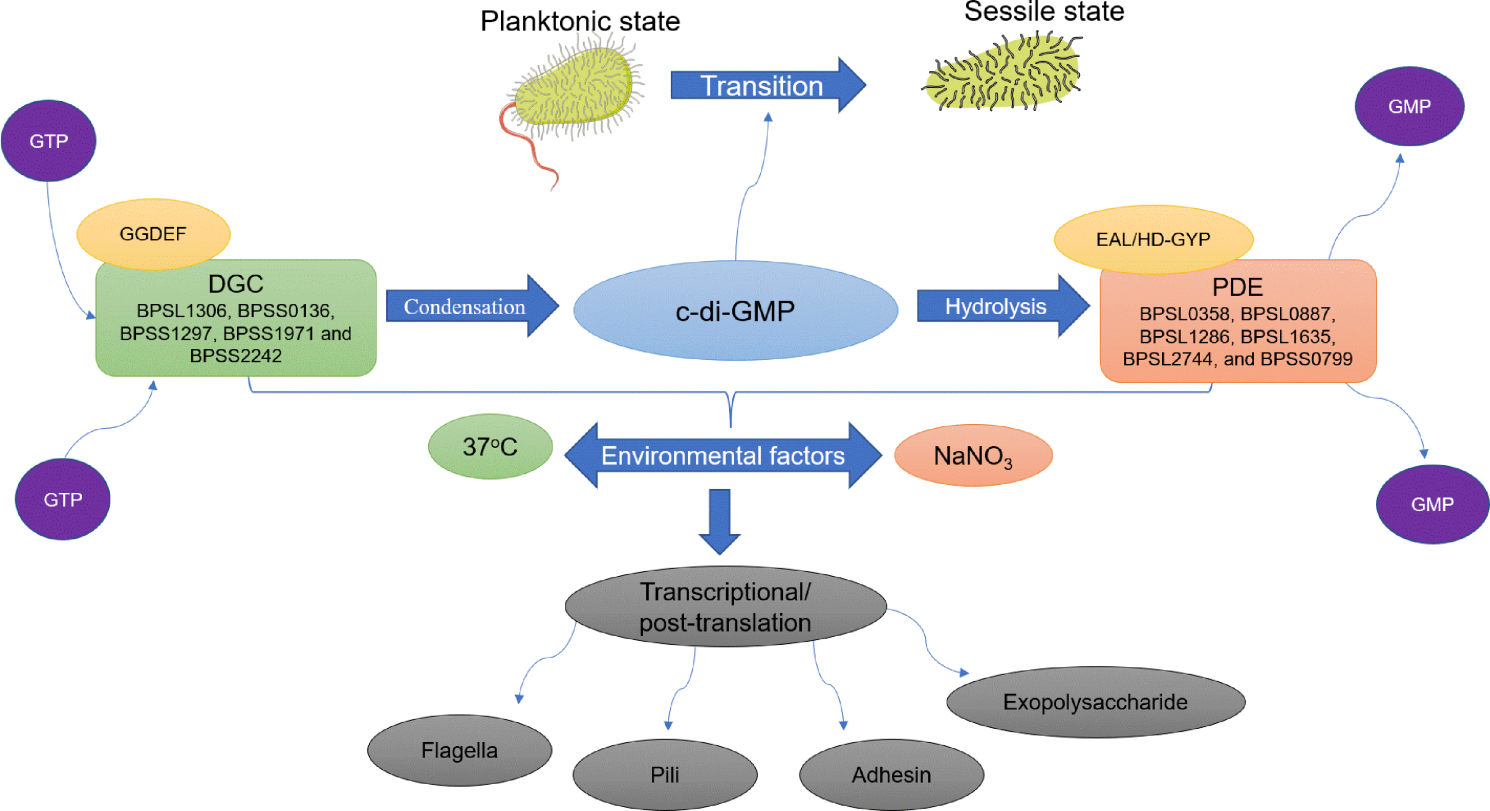
C-di-GMP synthesis mechanism and functional properties during BP biofilm formation. The synthesis and breakdown of cyclic-di-GMP (c-di-GMP) are regulated by two enzymes, diguanylate cyclase (DGC) and phosphodiesterase (PDE), each containing a conserved GGDEF or EAL/HD-GYP domain respectively. Two guanosine-5’-triphosphate (GTP) molecules are utilized by DGC during the condensation reaction that results in the formation of c-di-GMP, which favors biofilm formation by enhancing the transition from free-living planktonic cells to sessile cells. PDE catalyzes the hydrolysis of c-di-GMP into two guanosine monophosphate (GMP) molecules. Both enzymes are influenced by environmental signals such as temperature and concentration of sodium nitrate (NaNO_3_) that ultimately determine the level of c-di-GMP. The phenotypic characteristics of the cells such as the presence of flagella, pili, adhesin, and exopolysaccharide may be regulated by these enzymes at the transcriptional and post-translation levels through determining the level of c-di-GMP [41].

Several genes encoding proteins that contain the conserved GGDEF and EAL domains have been annotated in the BP genome (https://www.burkholderia.com/) and Plumley *et al*. [41, 54]. The proteins BPSL1306, BPSS0136, BPSS1297, BPSS1971, and BPSS2342 were predicted to carry the GGDEF domain with high sequence identity (Supplementary Fig. S1) while BPSL0358, BPSL0887, BPSL1286, BPSL1635, BPSL2744, and BPSS0799 have the EAL domain (Supplementary Fig. S2). Meanwhile, five other proteins (BPSL0602, BPSL1080, BPSL1263, BPSS0805, and BPSS2318) contain both the conserved GGDEF/EAL domains (Table 1) [41, 54]. By modulating the level of c-di-GMP, the GGDEF and EAL domains were reported to exert control at the transcriptional and post-translation level in regulating the expression of cell surface components (e.g., flagella, adhesins, pili, exopolysaccharides) (Fig. 2) [41]. Nitrate level and temperature have been proven to affect the DGC gene *Bp1026b_II2523* (*bpss2342*) and *cdpA*, respectively. Nonetheless, the function of other predicted genes that encode DGC and PDE enzymes containing GGDEF/EAL domains in BP needs to be further characterized in terms of their correlation to other specific environmental cues. It is known that proteins containing GGDEF/EAL domain generally assemble an N-terminal sensory domain which may respond to specific environmental stimuli (oxygen, light, nitric oxide, etc.) in regulating the enzyme activity that may determine the production level of c-di-GMP [55-57]. Based on currently available reports, we proposed the c-di-GMP synthesis mechanism and its functional properties during BP biofilm formation and the factors that may influence the biosynthesis of c-di-GMP in enhancing the transition from free-living planktonic cells to sessile cells, as illustrated in Fig. 2.

## Quorum Sensing (QS) Signaling

Quorum sensing is also a crucial signaling system involved in forming biofilms. Autoinducers produced by bacteria serve as chemical signal molecules and are released according to cell density [59, 60]. QS is utilized by both gram-positive and gram-negative bacteria [60]. In most *Burkholderia* spp., inhibition of this signaling system negatively affects biofilm formation, making the QS signaling system a suitable target for antimicrobials or anti-biofilm agents [61, 62].

*N*-acyl-homoserine lactones (AHLs) are the most common QS signaling molecule utilized by most gram-negative bacteria, including BP [63]. AHL signaling molecules are encoded by a class of genes that are homologous to the *luxI* and *luxR* of *Vibrio fischeri* and have been reported to mediate QS systems [65, 66]. *luxI* encodes AHL synthetases that are required for the synthesis of related signaling molecules, while LuxR family proteins serve as AHL molecular receptors [66, 67]. AHL autoinducers interact with the LuxR proteins to regulate the expression of genes that control relevant biological phenotypes, including biofilm formation [66, 67]. Similar QS systems in *Pseudomonas aeruginosa*, namely *lasIR* and *rhlRI*, are homologous to the LuxI-LuxR [68, 69]. In BP, the BpsI-BpsR QS system was reported as a homolog of LuxI-LuxR [70] and positively regulates biofilm formation.

BP owns three QS systems that produce AHL molecules, namely QS-1 (encoded by BpsI-BpsR), QS-2 (BpsI_2_-BpsR_2_), and QS-3 (BpsI_3_-BpsR_3_), which produce three types of AHLs, *N*-octanoylhomoserine lactone (C8HL), *N*-(3-hydroxy-octanoyl) homoserine lactone (OHC8HL), and *N*-(3-hydroxy-decanoyl) homoserine lactone [66, 71]. C8HL is synthesized by *N*-acyl-homoserine lactone synthase, encoded by *bpss0885* (*pmlI*), which is also known as *BpsI* [54, 66]. The remaining two AHLs are mainly produced by *BpsI_2_* (encoded by BPSS1180 in BP K96243) and *BpsI_3_* (BPSS1570), respectively, which are paralogs to *BpsI* [54, 66]. The expression of the three BpsI enzymes is regulated by their corresponding AHL- dependent transcription regulators BpsR, BpsR_2_, and BpsR_3_, respectively. BP strains lacking the BpsI-BpsR system cannot form biofilm [34, 66] while individual *bpsR* and *bpsI* mutants have impaired biofilm formation [66, 72]. Biofilm formation is restored in the presence of exogenous C8HL. In contrast, the addition of exogenous OHC8HL further suppresses biofilm formation in the mutant strains, indicating that exogenous OHC8HL serves as an antagonist in suppressing the biofilm formed by BP [66, 72]. Moreover, it was reported that BpsR_2_ is not involved in biofilm formation while BpsR_3_ plays a partial role. Unlike BpsR, exogenous OHC8HL was not able to resume full biofilm formation of BpsR_3_ mutant. Taken together, only dedicated QS signaling systems (QS-1 and QS-3) in BP were shown to be involved in biofilm formation, suggesting the specificity of AHL-signaling molecules in regulating the biofilm formation mechanism.

Apart from the AHL molecules, BP is known for producing another type of QS molecule known as 4-hydroxy-3-methyl-2-alkylquinolines (HMAQs), which are similar to the *Pseudomonas* quinolone signal (PQS), 4-hydroxy-2-alkylquinolines (HAQs) that are found in *P. aeruginosa* [73]. The PQS molecule is synthesized by the *pqsABCDE* operon (*pa0996*-*pa1000*) which is homologous to the *hhqABCDE* (*bpss0481*-*bpss0485*) genes in BP [73-75]. In *P. aeruginosa*, anthranilic acid is the precursor molecule for the synthesis of HAQs and is supplied by three different pathways that includes anthranilate synthase encoded by *phnAB* and *trpEG* and the degradation of tryptophan through the kynurenine pathway [75]. Similarly, BP produces anthranilic acid via the TrpEG and kynurenine pathway [75, 76]. In addition, inhibition of the kynurenine pathway was reported to increase the production of biofilm and reduce motility in BP [76], suggesting the involvement of HMAQs in biofilm formation and as a virulence factor of the bacterium.

In 2008, another quorum-sensing signal, cis-2-dodecenoic acid, also known as *Burkholderia* diffusible signal factor (BDSF), was reported in *B. cenocepacia* [77]. The BDSF QS system was reported to exert control towards AHL signaling and biofilm formation and affects the virulency of *B. cenocepacia* [78-81]. A *rpfF* gene that encodes RpfF_BC_ enzyme was found to be responsible for the synthesis of the BDSF, and the production of BDSF is regulated by the RqpSR two-component system [77, 82]. A neighboring gene of *rpfF*, namely *rpfR*, is a gene encoding protein containing a PAS-GGDEF-EAL domain associated with c-di-GMP synthesis. The deletion of *rpfR* resulted in increased intracellular c-di-GMP [80]. A further study shows that RpfR is a QS signal receptor that can interact with BDSF and a c-di-GMP phosphodiesterase that interacts with RpfF to inhibit BDSF production [83]. Moreover, RpfR can also act as a c-di-GMP sensor by interacting with the global regulator GtrR [83]. Interestingly, while homologs of RpfR, RqpSR two-component systems and GtrR were identified in BP, no RpfF_BC_ homologs could be detected [84]. However, there have yet to be any reports on RpfR, RqpSR two-component systems, and GtrR in BP. Therefore, it is unknown if the BDSF QS system that regulates c-di-GMP signaling exists in BP. Hence, further studies are warranted for a better understanding of the BDSF QS system and c-di-GMP in regulating the biofilm formation of BP.

## Regulation by Small RNAs (sRNAs)

sRNAs modulate protein expression by altering mRNA translation rates or via mRNA degradation [85]. Common metabolic processes regulated by sRNAs include QS, carbon metabolism, and iron homeostasis [86]. These metabolic processes were observed in a recent study on *B. cenocepacia* J3215 biofilm [85]. In addition, functional characterization of *B. cenocepacia* J3215 sRNAs through comparison between sRNA mutant and wild-type strains revealed high growth, cellular aggregation, and metabolic activity (upregulation of the tryptophan and phenylacetic acid degradation pathways) among the mutant strains [87]. A recent whole genome-level transcriptome study on *B. cenocepacia* J2315 biofilm and planktonic states highlighted the abundance of sRNAs in the biofilm transcriptome compared to bacteria in the planktonic state [85], thus suggesting that sRNAs may play a crucial role in the development of a successful biofilm. Fifteen of the identified sRNAs were highly conserved across *Burkholderia* spp. [85]. Nonetheless, to date, no biofilm-associated sRNAs have been described for BP. Therefore, further investigation to identify the presence and involvement of sRNAs is required to reach a better understanding of biofilm formation.

## Biofilm Composition in BP

The EPS matrix forms a natural protection shield for many bacteria, where it enables the bacteria that have changed from the planktonic stage growth mode to live in biofilm in response to various environmental cues and stresses. The formation and degradation of the EPS matrix in the biofilm life cycle are highly regulated and specific mechanisms are involved in the synthesis and degeneration of each of the EPS matrix components. Several major EPS matrix components in BP, including exopolysaccharides, eDNA, and proteins, have been identified. This section provides an overview of the three major EPS components of BP.

## Exopolysaccharide Biosynthesis

Exopolysaccharides are a major component of most bacterial biofilm matrices [40, 88, 89]. The exopolysaccharides have been categorized into various forms, such as capsular polysaccharides, free polysaccharides, and lipopolysaccharides (O-antigen) that have a key role in preventing the diffusion of antimicrobial agents within the biofilm community [89-91]. The exopolysaccharide in BP has been structurally classified to be acidic. It consists of a tetrasaccharide repeating unit composed of three galactose (with one bearing a 2-linked O-acetyl group) and a 3-deoxy-D-manno-2-octulosonic acid (KDO) residues ([→3)-β-D-Galp2Ac-(1→4)-A-D-Galp-(1→3)-β-D-Galp-(1→5)-β-Kdo-(2→]n) [92]. Later, glucose, mannose, and rhamnose were reported as the major type of monosaccharides predominantly found in BP biofilm exopolysaccharides [93]. While the chemical synthesis of the tetrasaccharide repeating unit of [→3)-β-D-Galp2Ac-(1→4)-A-D-Galp-(1→3)-β-D-Galp-(1→5)-β-Kdo-(2→] has been successfully carried out [94], the BP proteins that are responsible for the biosynthesis of KDO molecules remains unclear. A 3-deoxy-D-manno-octulosonate 8-phosphate phosphatase encoded by yrbI (*bpsl0537*) and responsible for hydrolysis of Kdo 8-phosphate to Kdo was found located in the operon *bpsl0534*-*bpsl0538* [54, 95]. In this operon, *bpsl0534* and *bpsl0536* encode the lipopolysaccharide export system ATP-binding proteins (ABC transporter), while *bpsl0535* and *bpsl0536* were annotated to encode an Ost-A-like protein and an arabinose-5-phosphate isomerase, respectively [54]. The involvement of the operon in the BP biofilm exopolysaccharide synthesis is yet to be investigated.

Recently, an exopolysaccharide gene cluster of 18 genes (*becA-R*) was identified. The *becA-R* is highly conserved within the *Burkholderia* spp. (*B. pseudomallei*, *B. thailandensis*, and *B. mallei*) [96]. The *becA-R* cluster encodes various enzymes such as glycosyl transferase, glycosyl hydrolase, capsular polysaccharide UDP-glucose lipid carrier transferase, and mannose-1-phosphate guanylyl transferase, which are required to synthesize exopolysaccharide components within the matrix [97, 98]. A transcriptome-level analysis of low and high BP biofilm producers revealed several genes within the *becA-R* gene cluster (*bpsl0603*, *bpsl0605*, *bpsl0618*, *bpsl0619*, and *bpsl0620*) were highly expressed in the high biofilm-producing strain [45]. Apart from the *becA-R* gene cluster, the wbiA gene cluster that consists of *bpsl2670* and *bpsl2671* was also identified to be involved in lipopolysaccharide biosynthesis. These genes encode UDP-glucose-4-epimerase and glycosyl transferase family protein, respectively [96]. Nonetheless, the detailed mechanism for exopolysaccharide biosynthesis has yet to be elucidated. Furthermore, several genes within the *becA-R* cluster encode hypothetical proteins, thus making elucidation of the exopolysaccharide synthesis mechanism more challenging.

Exopolysaccharide production in *Burkholderia* sp. biofilms is strongly influenced by c-di-GMP and QS signaling molecules [93, 97, 98]. The transcription regulation factors *bpsI*, *ppk* and *rpoS* were reported to influence the ratio of the monosaccharides glucose, galactose, mannose, and rhamnose of BP biofilm extracted exopolysaccharide [93]. In *B. cenocepacia*, c-di-GMP regulates exopolysaccharide biosynthesis at the post-translational level by promoting the binding between the CRP/FNR family transcriptional regulatory protein BCAM1349, (encoded by *bcam1349*) and the promoter region upstream of the *becA-R* gene cluster [97-99]. Two BP hypothetical proteins (BPSL0616 and BPSL0617) are reported to have a CRP/FNR superfamily domain, with BPSL0617 most likely an ortholog of BCAM1349 (Table 1) [45, 96].

**Fig. 3:**
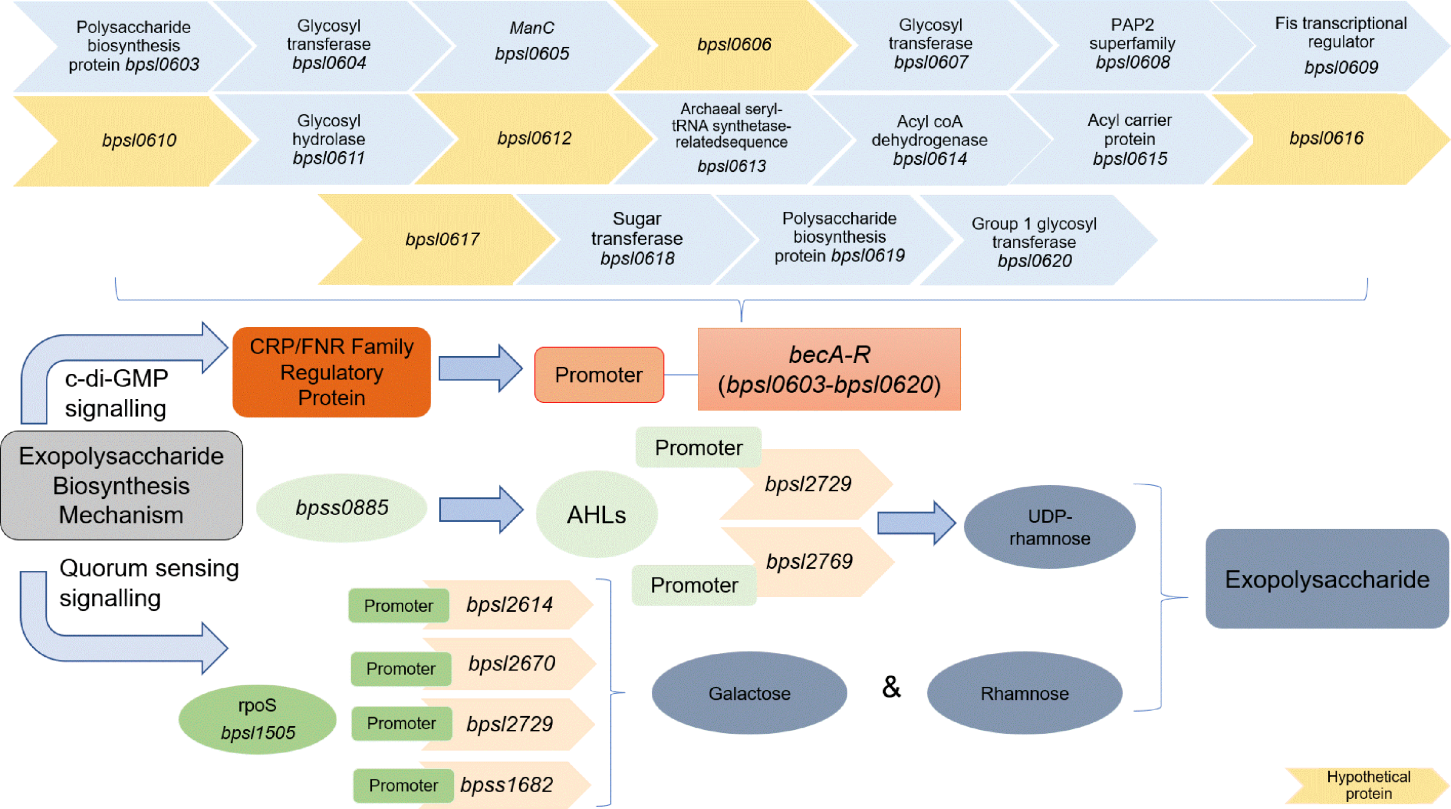
Proposed BP exopolysaccharide biosynthesis regulation mechanism via c-di-GMP and QS signaling. Signaling molecules, e.g. c-di-GMP, and QS molecules, e.g., RpoS and AHLs, regulate the development of the EPS components, particularly exopolysaccharides. c-di-GMP is reported to improve the binding between the regulatory protein and the promoter region of the *becA-R* gene cluster thereby triggering gene expression of the cluster to produce the enzymes that facilitate the synthesis of exopolysaccharides in the EPS.

Apart from c-di-GMP, *N*-acyl-homoserine lactone synthase BpsI (AHL synthase or C8HL, BPSS0885), the regulatory protein polyphosphate kinase (PPK, BPSL1366) and an alternative sigma factor S (RpoS, BPSL1505) are known to regulate exopolysaccharide production. Polyphosphate kinase is essential in producing inorganic polyphosphate from ATP which is required in the activation of sugar precursors for exopolysaccharide production [93]. A *bpsl1366* mutant showed increased susceptibility to antibiotics due to poor development of the exopolysaccharide framework [93]. AHL synthase and RpoS are crucial in regulating the expression of enzymes involved in the exopolysaccharide biosynthesis pathway to enhance the survival of biofilm cells under adverse conditions [93]. For example, UTP glucose-1-phosphate uridylyltransferase (BPSL2769) and GDP-mannose-4,6-dehydratase (WcbK) enzymes are involved in synthesizing UDP-rhamnose. The genes of these proteins are predicted to have a lux box promoter region that responds to BPSS0885 [93]. *rpoS* regulates a series of enzymes encoded by genes with an RpoS-dependent promoter region, such as glucokinase (BPSL2614), UDP-glucose 4-epimerase (BPSL2670), WcbK (BPSL2729), and UDP-glucose-1-phosphate uridylyltransferase (BPSS1682). These enzymes are involved in converting several monosaccharides into galactose and rhamnose [93]. The conversion of glucose into glucose-6-phosphate catalyzed by glucokinase is the first step in biofilm exopolysaccharide synthesis; this highlights the importance of *rpoS* in regulating exopolysaccharide synthesis in BP biofilms [93]. Furthermore, monosaccharides, particularly rhamnose, contribute to a robust biofilm matrix that significantly limits the diffusion of antibiotics [93]. Therefore, overexpression of *bpss0885*, *bpsl1505*, and *bpsl1366* accompanied by the accumulation of c-di-GMP, may lead to the formation of a rigid biofilm [93, 97]. The mechanisms of c-di-GMP, QS signaling, and RpoS involved in exopolysaccharide biosynthesis for BP biofilm formation are proposed and illustrated in Fig. 3.

## Extracellular DNA (eDNA) in EPS

Extracellular DNA (eDNA) is a crucial component of EPS and biofilm development [100-102]. eDNA is proposed as a key component of many pathogenic bacteria that form biofilms where it contributes to shielding biofilm against antimicrobial agents, promoting adhesion, and strengthening the integrity of biofilms [101, 103, 104]. In some bacteria, eDNA is derived from chromosomal DNA that is released from the bacterial cells either by active secretion mediated by QS or through cell lysis [105-107]. These mechanisms of eDNA release have been widely described for *Staphylococcus epidermidis* and *P. aeruginosa* biofilms [108, 109] but are yet to be characterized for BP. However, there is some indication that eDNA production in BP occurs through the extrusion of DNA from living cells, which is controlled by the transcriptional regulator BPSL1887 [110]. Interestingly, recent studies aiming to determine and quantify the components of BP and *B. thailandensis* biofilms revealed that eDNA and other major components in the biofilms are synthesized by living cells [111]. In addition, strains lacking capsular polysaccharides (CPS I) compensate by producing high levels of eDNA to complete biofilm formation with the abundant eDNA contributing to the thickness of the biofilm matrix [111]. BP *bpsl1036* and *bpsl1037* mutants that lack the two-component signal transduction systems (TCSTS) implicated in virulence and drug resistance have increased eDNA levels which ultimately promotes biofilm formation in BP [112].

It was reported that eDNA is actively involved during the early stages of biofilm formation, facilitating initial attachment and bacterial aggregation under the planktonic and biofilm states [113, 114]. Deoxyribonucleases (DNAses) are able to completely inhibit eDNA activity which is reflected by a reduced biofilm mass. However, inhibition of eDNA activity beyond the initial biofilm formation step shows no significant changes in biofilm mass, due to limited access of DNAse towards eDNA in mature biofilm. Therefore, DNAse treatment could be an appropriate treatment strategy targeting eDNA during the early stages of biofilm infections [113]. The ability of eDNA to defend the biofilm community against antimicrobial agents arises from its chemical properties. The negatively charged eDNA binds to the positively charged ions on antibiotics such as aminoglycosides and antimicrobial peptides, thereby reducing the antimicrobial agents’ efficiency in eliminating biofilm-forming pathogens [100, 115]. When BP biofilm was subjected to DNase treatment, a drastic reduction in biofilm mass was observed which could not be restored following supplementation with exogenous DNA [113]. A similar observation was noted with *Neisseria meningitidis* [116], and taken together, implies the importance of BP eDNA in the formation of BP biofilms. More recently, the unraveling of the *P. aeruginosa* eDNA structure revealed that it was different from *P. aeruginosa* chromosomal DNA where purine-rich RNAs that were integrated into the eDNA framework enabled crosslinking of the extracellular matrix [117]. Moreover, the formation of G-quadruplexes occurs in eDNA due to the non-canonical Hoogsteen base pairing between thymine/uracil and guanine [118]. The presence of G-quadruplexes in the matrix of *P. aeruginosa* biofilms was verified by specific antibody binding while the loss of the G-quadruplexes resulted in a lack of eDNA fibers [118]. Hence, this marks the structural specificity of eDNA involved in biofilm formation.

eDNA also exists as a lattice structure stabilized by DNABII proteins [119]. The integration host factor (IHF) and histone-like protein (HU) are two common members of the DNABII protein family that contribute to the lattice structure of the eDNA, thereby increasing the structural stability of the biofilm [120-122]. The *B. cenocepacia* HU and IHF protein orthologs are present in BP [122] (Table 2), indicating similar structural integrity components among the *Burkholderia* biofilms. Targeting the DNABII proteins via anti-DNABII antibodies effectively reduced biofilm formation in *P. aeruginosa* 27853, *B. cenocepacia* K56, non-typeable *Haemophilus influenzae* 86-028NP, *Moraxella catarrhalis* 7169, and *Staphylococcus aureus* 29213 [123]. These findings highlight the significance of eDNA in the structural integrity of biofilms for most bacteria. Therefore, targeting the eDNA could be a therapeutic strategy to eradicate infections by biofilm-forming pathogens.

## Proteins in EPS

The abundance of proteins in EPSs has been examined recently in most bacteria capable of forming biofilms. The function of these proteins to achieve a successful biofilm are diverse [124]. Currently, proteins within EPSs are categorized as enzymes and structural proteins [125]. Numerous enzymes in EPSs are involved in the synthesis or degradation of matrix components. For instance, tyrosine kinase encoded by *bceF* has been implicated in favoring biofilm formation by mediating the synthesis of exopolysaccharides in *B. cepacia* IST408 [126]. On the contrary, enzymes that break down the EPS, such as alginate lyase, are involved in the breakdown of exopolysaccharides in *P. aeruginosa* biofilms [127]. The BP genes encoding tyrosine kinase and alginate lyase annotated in the *Burkholderia* Genome Database (https://www.burkholderia.com/) are shown in Table 2 [54]. Further investigation is required to assess the enzymatic activity of these enzymes towards the biofilm formed by BP.

EPS proteins that contribute to structural stability include surface-associated proteins, such as pili and flagella, which mediate bacterial initial attachment and adhesion in *H. influenzae* biofilms and most other bacterial biofilms [124, 128]. Pili are known to facilitate bacterial adhesion, motility, DNA transfer, and biofilm formation [129]. BP is known to encode eight types of type IV pili (T4P) [130]. Recently, an uncharacterized type IV pili-associated protein (TFP8) encoded by *bpss2185* was reported to be highly expressed during biofilm maturation and dispersal stages, highlighting that bacterial movement is crucial in stabilizing the structure of biofilm in BP [131]. Apart from that, proteomics analysis had discovered an abundance of outer membrane vesicle (OMV) proteins within the matrix of *P. aeruginosa* and *B. multivorans* biofilms [102]. The OMV proteins of gram-negative bacteria exist as spherical and bilayer membranes [132]. OMVs released during bacterial growth and and contain lipoproteins, lipopolysaccharides, and outer membrane proteins [133, 134]. OMVs are involved in several phenomena such as pathogenesis, bacterial communication, horizontal gene transfer, nutrient capture, bacterial-host interaction, and improvement of coaggregation during biofilm formation [102, 134]. Furthermore, OMVs can shield the biofilm community by releasing toxins that can target and affect the host defensive responses [133]. Meanwhile, *bpss0093* and *bpsl1800*, two BP genes, were reported to be highly expressed during biofilm formation [45]. These genes are suggested to encode an outer membrane usher protein, presumably with a similar function to OMVs. Since the abundance of OMVs has been reported within the EPS, utilizing these OMVs to channel the antibacterial agents into the biofilm community serves as a strategy to eradicate biofilm infection [133].

## Conclusion and Future Perspective

BP biofilms have been implicated as a virulence factor contributing to the pathogenesis of melioidosis during BP infections. This review systematically presents the genes and proteins that have been shown or predicted to be involved in the biosynthesis of essential *B. pseudomallei* EPS components. More than 60 genes and proteins representing 1.2% of the total annotated genes of BP have been identified as being involved in its biofilm formation. In this review, we have highlighted several knowledge gaps that require future investigation. These include: (i) the need to elucidate the roles of putative proteins that contain DGC and PDE domains for cyclic-di-GMP signaling; (ii) the determination of specific sRNAs that may have roles in regulating BP biofilm formation;(iii) the characterization of enzymes including hypothetical proteins in the *becA-R* gene cluster to decode the exopolysaccharide biosynthesis pathway; and (iv) to unravel the mechanistic role of eDNA in biofilm formation and its potential as a target for therapeutics. Furthermore, a systems biology approach could be adopted to characterize further the interrelationship between biofilm formation stages, signaling systems, regulation, and biosynthesis of EPS components.

## Supplemental Materials

Supplementary data for this paper are available on-line only at http://jmb.or.kr.

## Figures and Tables

**Table 1:** Proteins of *B. pseudomallei* that are involved in signaling system in the regulation of biofilms.

Signaling Molecules	Annotation/Description	Species/isolate	Sequence identity to K96243 (% identity)	*Burkholderia pseudomallei* K96243 identifier code	Protein Description for *Burkholderia pseudomallei* K96243	Reference
c-di-GMP	*Bp1026b_II2523* DGC	*Burkholderia pseudomallei* *1026b*	99.89	BPSS2342	Hypothetical protein	[41,54]
	*Bp1026b_I2235* GGDEF domain	*Burkholderia pseudomallei* *1026b*	99.85	BPSL1306	Hypothetical protein	[41,54]
	*Bp1026b_II0153* GGDEF domain	*Burkholderia pseudomallei* *1026b*	99.93	BPSS0136	Hypothetical protein	[41,54]
	*Bp1026b_II1380* GGDEF domain	*Burkholderia pseudomallei* *1026b*	99.73	BPSS1297	Regulatory protein	[41,54]
	*Bp1026b_II2115* GGDEF domain	*Burkholderia pseudomallei* *1026b*	99.87	BPSS1971	Two-component system fusion protein	[41,54]
	*Bcam2836* putative DGC	*Burkholderia cenocepacia* *J2315*	85.70	BPSS2342	Hypothetical protein	[51,54]
	*BTH_II2363* (*pdcA*) GGDEF domain	*Burkholderia thailandensis* *E264*	97.43	BPSS2342	Hypothetical protein	[53,54]
	*BTH_II2364* (*pdcB*) CheC/CheX domain	*Burkholderia thailandensis* *E264*	98.52	BPSS2343	Hypothetical protein	[53,54]
	*BTH_II2365* (*pdcC*) phosphate-accepting response regulator	*Burkholderia thailandensis* *E264*	96.72	BPSS2344	Hypothetical protein	[53,54]
	*Bp1026b_I0571* EAL domain	*Burkholderia pseudomallei* *1026b*	99.88	BPSL2744	Hypothetical protein	[41,54]
	*Bp1026b_I1579* EAL domain	*Burkholderia pseudomallei* *1026b*	100	BPSL1635	Hypothetical protein	[41,54]
	*Bp1026b_I2260* EAL domain	*Burkholderia pseudomallei* *1026b*	99.38	BPSL1286	Hypothetical protein	[41,54]
	*Bp1026b_I2659* EAL domain	*Burkholderia pseudomallei* *1026b*	99.53	BPSL0887	Hypothetical protein	[41,54]
	*Bp1026b_I3148* EAL domain	*Burkholderia pseudomallei* *1026b*	99.84	BPSL0358	Hypothetical protein	[41,54]
	*Bp1026b_II0879* EAL domain	*Burkholderia pseudomallei* *1026b*	99.48	BPSS0799	Hypothetical protein	[41,54]
	*BCAL0652* EAL domain	*Burkholderia cenocepacia* *J2315*	30.17	BPSL2744	Hypothetical protein	[51,54]
	*Bp1026b_I2284* (*CdpA*) GGDEF/EAL domain	*Burkholderia pseudomallei* *1026b*	99.95	BPSL1263	Hypothetical protein	[41,42,54]
	*BCAL1069* (*cdpA*) GGDEF/EAL domain	*Burkholderia cenocepacia* *J2315*	85.52	BPSL1263	Hypothetical protein	[51,54,135]
	*Bp1026b_I2456* GGDEF/EAL domain	*Burkholderia pseudomallei* *1026b*	99.79	BPSL1080	Hypothetical protein	[41,54]
	*Bp1026b_I2928* GGDEF/EAL domain	*Burkholderia pseudomallei* *1026b*	99.40	BPSL0602	Hypothetical protein	[41,54]
	*Bp1026b_II0885* GGDEF/EAL domain	*Burkholderia pseudomallei* *1026b*	99.71	BPSS0805	Hypothetical protein	[41,54]
	*Bp1026b_II2498* GGDEF/EAL domain	*Burkholderia pseudomallei* *1026b*	99.83	BPSS2318	Hypothetical protein	[41,54]
	*Bcam1160* putative c-di-GMP	*Burkholderia cenocepacia*	86.75	BPSL1080	Hypothetical protein	[51,54]
	*Bcam1349* CRP/FNR family transcriptional regulator	*Burkholderia cenocepacia* *J2315*	79.07	BPSL0617	Hypothetical protein	[45,54,96,98]
	CRP/FNR superfamily	*Burkholderia pseudomallei* *K96243*	NA	BPSL0616	Hypothetical Protein	[45]
QS	*BpsI* autoinducer synthase	*Burkholderia pseudomallei* *K96243*, *KHW, H11*	100	BPSS0885 BPSS1570	N-acyl-homoserine lactone synthase	[54,66]
	*BpsR* autoinducer binding transcriptional regulator	*Burkholderia pseudomallei* *K96243*, *KHW, H11*	99.86	BPSS0887	N-acyl-homoserine lactone dependent regulatory protein	[54,66]
	*PA0996* (*pqsA*)	*Pseudomonas aeruginosa* *PAO1*	30.36	BPSS0481	HhqA	[54,73-75,136]
	*PA0997* (*pqsB*)	*Pseudomonas aeruginosa* *PAO1*	38.32	BPSS0482	HhqB	[54,73-75,136]
	*PA0998* (*pqsC*)	*Pseudomonas aeruginosa* *PAO1*	38.59	BPSS0483	HhqC	[54,73-75,136]
	*PA0999* (*pqsD*)	*Pseudomonas aeruginosa* *PAO1*	53.68	BPSS0484	HhqD	[54,73-75,136]
	*PA1000* (*pqsE*)	*Pseudomonas aeruginosa* *PAO1*	30.36	BPSS0485	HhqE	[54,73-75,136]

*NA- Not applicable

**Table 2:** Genes/proteins involved in the contribution of extracellular polymeric matrix (EPS) components in *B. pseudomallei* biofilms.

EPS components	Gene/gene cluster reported to be involved in EPS biosynthesis (Annotation/Description)	Species/isolate	Sequence identity to BP K96243 (% of identity)	*Burkholderia pseudomallei* K96243 identifier code	Protein Description of *Burkholderia pseudomallei* K96243	Reference
Exopolysaccharide	*Bcam1330* (putative exopolysaccharide export protein)	*Burkholderia cenocepacia* *J2315*	79.10	BPSL2780	Capsular polysaccharide transport protein	[54,97]
	*Bcam1331* (putative tyrosine kinase protein)	*Burkholderia cenocepacia* *J2315*	-	-	-	[54,97]
	*Bcam1332* (hypothetical protein)	*Burkholderia cenocepacia* *J2315*	-	-	-	[54,97]
	*Bcam1333* (putative exopolysaccharide acyltransferase)	*Burkholderia cenocepacia* *J2315*	73.68	BPSL3087	Acyltransferase	[54,97]
	*Bcam1334* (hypothetical protein)	*Burkholderia cenocepacia* *J2315*	70.37	BPSL0610	Hypothetical protein	[54,97]
	*Bcam1335* (glycosyltransferase)	*Burkholderia cenocepacia* *J2315*	71.77	BPSL0604	Glycosyltransferase	[54,97]
	*Bcam1336* (putative exopolysaccharide transporter)	*Burkholderia cenocepacia* *J2315*	74.91	BPSL0603	polysaccharide biosynthesis protein	[54,97]
	*Bcam1337* (glycosyltransferase)	*Burkholderia cenocepacia* *J2315*	-	-	-	[54,97]
	*Bcam1338* (glycosyltransferase)	*Burkholderia cenocepacia* *J2315*	-	-	-	[54,97]
	*Bcam1339* (hypothetical protein)	*Burkholderia cenocepacia* *J2315*	73.68	BPSL1233	Lipoprotein	[54,97]
	*Bcam1340* (mannose-1-gyanylyltransferase)	*Burkholderia cenocepacia* *J2315*	83.60	BPSL0605	Mannose-1-phosphate guanylyltransferase (*manC*)	[45,54,97]
	*Bcam1340* (mannose-1-gyanylyltransferase)	*Burkholderia cenocepacia* *J2315*	74.61	BPSS1835	LPS biosynthesis mannose-1-phosphate guanylyltransferase (*BceA*)	[54,97]
	*Bcam1341* (hypothetical protein)	*Burkholderia cenocepacia* *J2315*	77.67	BPSL0606	Hypothetical protein	[54,97]
	-	*Burkholderia pseudomallei* *K96243*	NA	BPSL0618	putative sugar transferase	[45]
	-	*Burkholderia pseudomallei* *K96243*	NA	BPSL0619	putative polysaccharide biosynthesis/export protein	[45]
	-	*Burkholderia pseudomallei* *K96243*	NA	BPSL0620	glycosyl transferase group 1 protein	[45]
	-	*Burkholderia pseudomallei* *K96243*	NA	BPSS1649	sugar-binding protein	[45]
	-	*Burkholderia pseudomallei* *K96243*	NA	BPSS1978	EPS transport-related membrane protein kinase	[45]
	*Bp 1026b_I2907-Bp1026b_I2927* *becA-R*	*Burkholderia pseudomallei* *1026b*	NA	BPSL0603-BPSL0620	Exopolysaccharide gene cluster	[96]
	*Bp1026b-I0648 wbiA*	*Burkholderia pseudomallei* *1026b*	99.1	BPSL2671	Glycosyltransferase family protein	[54,96]
	*Bp1026b-I0649 wbiA*	*Burkholderia pseudomallei* *1026b*	100	BPSL2670	UDP-glucose-4-epimerase	[54,96]
	*bps*I	*Burkholderia pseudomallei* *K96243*	NA	BPSS0885	acyl homoserine lactone (AHL)	[93]
	*rpo*S	*Burkholderia pseudomallei* *K96243*	NA	BPSL1505	RNA polymerase sigma factor	[93]
	-	*Burkholderia pseudomallei* *K96243*	NA	BPSL1366	polyphosphate kinase	[93]
	*wcbK*	*Burkholderia pseudomallei* *K96243*	NA	BPSL2729	UTP glucose-1-phosphate	[93]
eDNA	Bcal1585 (histone like protein) (hupb)	*Burkholderia cenocepacia* *J2315*	76.98	BPSL0004	DNA-binding protein HU-alpha	[54,122]
	Bcal3530 (histone like protein) (hupA)	*Burkholderia cenocepacia* *J2315*	93.45	BPSL0004	DNA-binding protein HU-alpha	[54,122]
	Bcal1487 (integration host factor alpha)	*Burkholderia cenocepacia* *J2315*	88.04	BPSL1939	integration host factor alpha	[54,122]
	Bcal2949 (integration host factor beta)	*Burkholderia cenocepacia* *J2315*	89.56	BPSL2514	integration host factor beta	[54,122]
	BPSL1887 (transcriptional regulatory protein)	*Burkholderia pseudomallei* *K96243*	NA	BPSL1887	sigma-54 related transcriptional regulatory protein	[110]
Proteins	-	*Burkholderia pseudomallei* *K96243*	NA	BPSS0093	outer membrane usher protein	[45]
	-	*Burkholderia pseudomallei* *K96243*	NA	BPSL1800	outer membrane usher protein	[45]
	*bceF*	*Burkholderia pseudomallei* *K96243*	NA	BPSS1830	Tyrosine kinase	[54]
	AK34_RS27645 (Alginate lyase)	*Burkholderia dolosa AU0158*	85.42	BPSL3363	Hypothetical protein	[54]
	-	*Burkholderia pseudomallei* *K96243*	NA	BPSL0782	Type 4 Pili 1	[130]
	-	*Burkholderia pseudomallei* *K96243*	NA	BPSL1821	Type 4 Pili 2	[130]
	-	*Burkholderia pseudomallei* *K96243*	NA	BPSL1899	Type 4 Pili 3	[130]
	-	*Burkholderia pseudomallei* *K96243*	NA	BPSL2752	Type 4 Pili 4	[130]
	-	*Burkholderia pseudomallei* *K96243*	NA	BPSL2756	Type 4 Pili 4	[130]
	-	*Burkholderia pseudomallei* *K96243*	NA	BPSL3008	Type 4 Pili 5	[130]
	-	*Burkholderia pseudomallei* *K96243*	NA	BPSL3170	Type 4 Pili 6	[130]
	-	*Burkholderia pseudomallei* *K96243*	NA	BPSS1593	Type 4 Pili 7	[130]
	-	*Burkholderia pseudomallei* *K96243*	NA	BPSS1595	Type 4 Pili 7	[130]
	-	*Burkholderia pseudomallei* *K96243*	NA	BPSS2185	Type 4 Pili 8	[130]
	-	*Burkholderia pseudomallei* *K96243*	NA	BPSS2186	Type 4 Pili 8	[130]

*NA-Not applicable
