# Supplementary material for: Biofilm Signaling, Composition and Regulation in Burkholderia pseudomallei
Source: J Microbiol Biotechnol. 2022 Oct 17;33(1):15–27. doi: 10.4014/jmb.2207.07032 (PMC9899790; doi:10.4014/jmb.2207.07032)
Supplement: Supplementary file 1 [file jmb-33-1-15-supple.pdf]

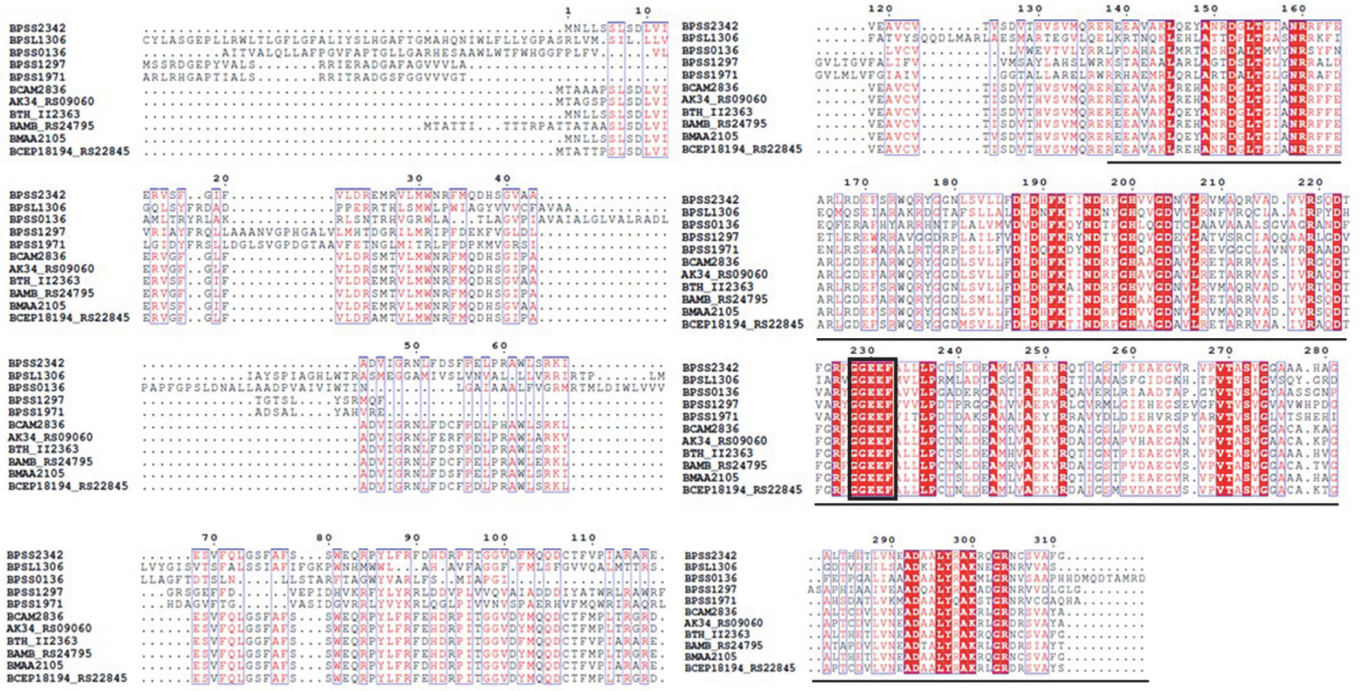

Supplementary Figure 1: Multiple Sequence alignment (MSA) of diguanylate cyclase (DGC), BPSS2342 against proteins with conserved GGDEF domain (underlined) in BP (BPSL1306, BPSS0136, BPSS1297, BPSS1971), ***B. cenocepacia*** **(BCAM2836),** ***B. dolosa*** **(AK34_RS09060),** ***B. thailandensis*** **(BTH_II2363),** ***B. ambifaria*** **(BAMB_RS24795),** ***B. mallei*** **(BMAA2105) and** ***B. lata*** **(BCEP18194_RS22845). Fully conserved regions are highlighted in red while partially conserved sequences are represented with a box. A conserved motif of GGEEF (black box) was observed in the GGDEF domain. The sequences were aligned with Clustal Omega [137] and visualised using ESPript 3.0 [138].**

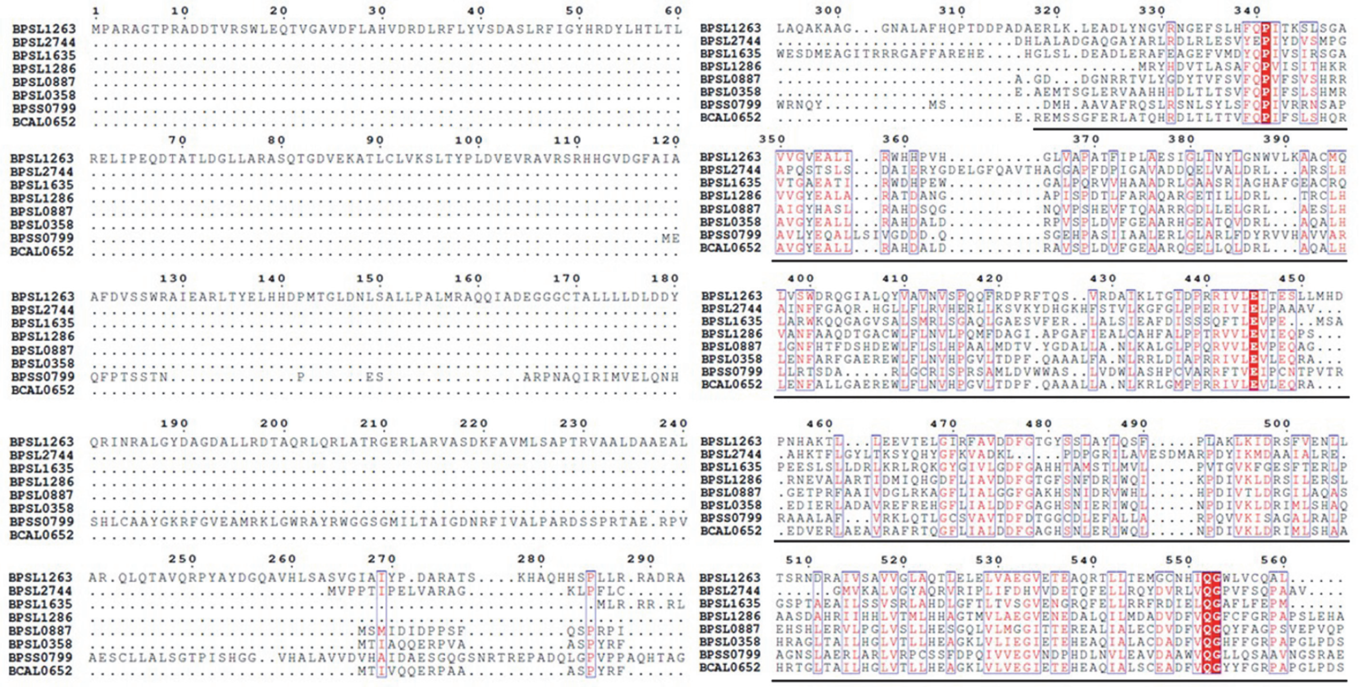

Supplementary Figure 2: Multiple Sequence alignment (MSA) of predicted phosphodiesterase (PDE) that contains conserved EAL/HD-GYP domain (underlined) in ***B. pseudomallei*** **(BPSL1263, BPSL2744, BPSL1635, BPSL1286, BPSL0887, BPSL0358 and BPSS0799), and** ***B. cenocepacia*** **(BCAL0652). The sequences were aligned with Clustal Omega [137] and visualized using ESPript 3.0 [138].**
